# A Scoping Review of West Nile Virus Seroprevalence Studies among African Equids

**DOI:** 10.3390/pathogens10070899

**Published:** 2021-07-15

**Authors:** Olaolu T. Olufemi, Marta Barba, Janet M. Daly

**Affiliations:** 1 One Virology—Wolfson Centre for Global Virus Research, School of Veterinary Medicine and Science, University of Nottingham, Loughborough LE12 5RD, UK; olaolu.olufemi@nottingham.ac.uk; 2 Department of Veterinary Public Health and Preventive Medicine, Faculty of Veterinary Medicine, University of Jos, Jos 930003, Nigeria; 3 Microbiological Agents Associated with Animal Reproduction (ProVaginBio), Veterinary Faculty, University Cardenal Herrera-CEU, CEU Universities, 46115 Alfara del Patriarca, Spain; martabrvet@gmail.com; 4 Innovation Centre in Rural Bioeconomy, Agrifood Research and Technology Centre of Aragon (CITA), Av. Montañana, 930, 50059 Zaragoza, Spain

**Keywords:** West Nile virus, flavivirus, antibodies, Africa, horses, donkeys, mules, seroprevalence

## Abstract

West Nile virus (WNV) is an emerging and re-emerging zoonotic flavivirus first identified in and endemic to Africa. The virus is transmitted between birds by biting mosquitoes, with equids and humans being incidental hosts. The majority of infected incidental hosts display no or only mild clinical signs, but a fraction develop encephalitis. The aim of this scoping review was to identify and evaluate primary research on the presence of antibodies to WNV among African equids. Three bibliographic databases and the grey literature were searched. Of 283 articles identified, only 16 satisfied all the inclusion criteria. Data were collated on study design and outcomes. The overall seroprevalence reported ranged from 17.4 to 90.3%, with 1998 (35%) of the 5746 horses, donkeys and mules having screened positive for WNV antibodies. Several articles determined that seroprevalence increased significantly with age. Due to co-circulation of other flaviviruses in Africa, in the majority of studies that screened samples by ELISA, positive results were confirmed using a more specific neutralization test. However, only eight studies tested against other flaviviruses, including Potiskum, Uganda S, Wesselsbron and yellow fever virus in one, Japanese encephalitis and Usutu virus (USUV) in one, tick-borne encephalitis and USUV in one and USUV only in three. Equids are regarded as useful sentinel animals for WNV, but variation in study design poses challenges when trying to determine risk factors for, and trends in, WNV seroprevalence.

## 1. Introduction

West Nile virus (WNV) is a mosquito-borne virus that affects humans and equids. It belongs to the Japanese encephalitis virus (JEV) sero-complex group within the family *Flaviviridae* and genus *Flavivirus* [1]. The virus was first isolated from humans in 1937, specifically from the blood of a woman who suffered mild febrile illness in the West Nile province of Uganda [2], and from horses in 1963 [3]. West Nile virus is believed to be the most pervasive arbovirus, with impacts on animal and human health [4]. The disease now has an almost worldwide distribution across Africa, Asia, Australia, the Middle East, North America and Europe [5,6,7]. Wild birds serve as primary reservoir hosts and the enzootic transmission cycle is maintained by infected *Culex* spp. mosquitoes [5]. Horses are excellent sentinels for WNV and can be used to forecast WNV risk patterns in humans [8]. Equids are more predisposed to WNV than humans, with possible manifestation of nervous signs in around 10% of infected horses [9].

West Nile virus is endemic to Africa, which is probably the source of all its lineages and genotypes [10]. The continual emergence and re-emergence of WNV in parts of Africa pose veterinary and public health threats due to the resultant increased morbidity and mortality among humans, horses and birds [11,12]. The abundance of wild birds and ornithophilic mosquitoes on the African continent provides a suitable scenario for WNV transmission to horses and other susceptible hosts. Migratory birds are abundant in Africa [13,14] and their migratory routes play a significant role in the transmission of WNV [15]. Climate change has greatly influenced the expansion of vectors that may transmit WNV and other vector-borne diseases from endemic to non-endemic environments [16]. Human and animal health are largely impacted negatively by climate change, especially in low-income counties [17].

Transient and low-level viremia produced by WNV limits the use of RT-PCR for molecular diagnosis [18,19,20,21]. While antibody testing is extensively used for WNV diagnosis, cross reactivity with other flaviviruses makes diagnosis based on immunoglobulin M (IgM) and IgG detected by enzyme-linked immunosorbent assay (ELISA) alone unreliable [22]. Neutralization tests, which are more specific, are considered the gold standard for WNV diagnosis and are used to validate ELISA results [23,24,25].

Although the disease has been reported in horses in a few countries in Africa, there is no published study on the prevalence of antibodies to WNV in equids across Africa or the drivers of its emergence. The aim of this scoping review was to integrate the existing data on the prevalence of antibodies to WNV among African equids. The primary objective was to evaluate the reliability of what is known about the seroprevalence of WNV among equids in different regions of Africa. A further objective was to determine what is known about risk factors associated with the emergence of the disease in these regions. This scoping review provides veterinarians and agencies with an overview of the existing literature on the prevalence of WNV antibodies in horses, including providing evidence on the burden of WNV in African equids and highlighting knowledge gaps, thereby giving direction to future surveillance efforts.

## 2. Results

### 2.1. Literature Search

**Figure 1:**
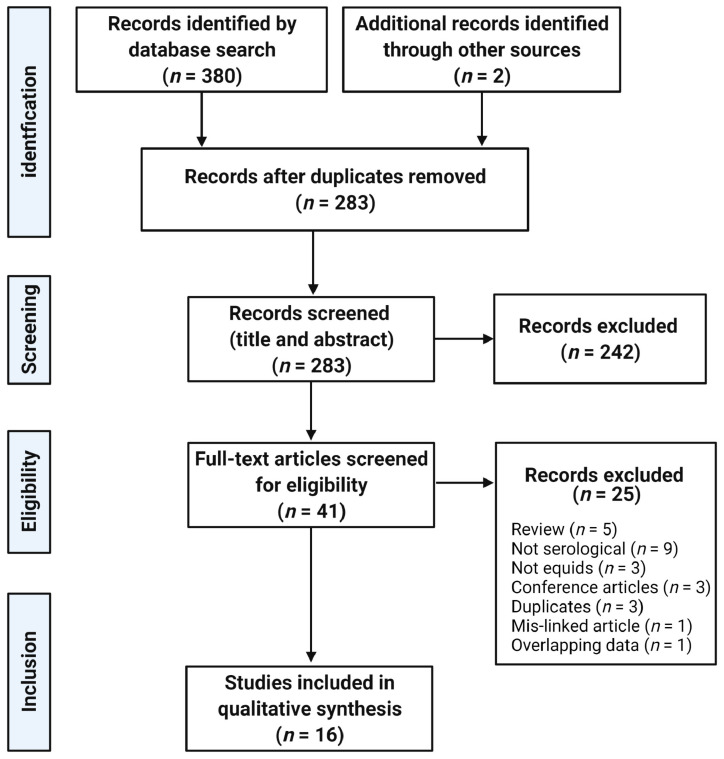
PRISMA flow diagram of literature search and screening articles. (Created with BioRender.com, accessed on 10 July 2021).

A total of 283 unique articles from an initial database search of 382 were screened. Title and abstract screening resulted in the exclusion of 242 articles. Of the 41 full-text articles assessed for eligibility, 25 were excluded with reasons listed in Figure 1. Finally, 16 articles satisfied the inclusion criteria.

### 2.2. Study Characteristics

Most studies involved samples from a single country (Table 1) and were unevenly distributed across the continent. Eight studies (50%) were from North Africa only while five studies (31.3%) were from West Africa. One study (6.3%) from East Africa involved four islands: Madagascar, Mauritius, Réunion and Seychelles. There was no independent study in Central Africa although one study involved equids in West, East and Central African countries. Only one study (6.3%) was conducted in the South African region. Of the 16 articles included, 15 (93.8%) were published since 2000 (Table 1).

Study designs included two cohort studies, while others were cross-sectional and one was descriptive (Table 1). Most studies used non-probability sampling techniques, including convenience and purposive/judgmental. In a few studies, probability sampling techniques were used, including random, cluster and multistage sampling. All equids of volunteer owners were sampled in Northwest Senegal and every horse available without WNV vaccination was sampled in Southwestern Nigeria. Two recent studies conducted a survey of vectors alongside the seroprevalence of WNV in equids [26,40].

Sample size varied from 62 to 1189 equids (Table 2). Only three studies (18.8%) presented a sample size calculation for the equids to be sampled. A total of 5746 equids comprising of 4323 (75.2%) horses, 1277 (22.2%) donkeys, 144 (2.5%) mules and 2 (0.03%) unspecified equids were screened across all the studies selected for this scoping review (Table 2). Eleven (68.8%) studies investigated antibodies to WNV in horses only while others also screened donkeys, mules, dogs, cattle, sheep and goats as well as unstated equids. Most studies screened equids older than 2 years with an overall range of 1–32 years. Four studies did not give an age description of the equine population, while two gave a general description of ‘adults’. Whether male and/or female equids were included was only stated in half of the studies and the proportions of each was not revealed or was unclear in some. Thoroughbred, Argentine criollo, Dongola, Arab-Barb, European and Moroccan studs, local and mixed breeds were among the breeds screened for antibodies to WNV, although breed was not mentioned in most of the studies (Table 2).

### 2.3. Seroprevalence

A wide range of assays was used to screen equids for antibodies to WNV across studies (Table 3). The notable reported assays were IgG enzyme-linked immunosorbent assay (ELISA), either in-house, commercial, antigen capture, competitive (cELISA) or IgM. Other tests used included micro-immunoassay, Western blot and neutralization tests, plaque reduction neutralization (PRNTs) (*n* = 7), virus neutralization (*n* = 2) and micro-neutralization (*n* = 2). Two studies did not confirm the results of the samples screened by cELISA. One study detected antibodies against non-structural 1 protein using an in-house ELISA to differentiate between infected and vaccinated horses. Most assays were combined with other methods except the complement fixation test (CFT) used in the earliest study.

The virus used for the neutralization test was clearly stated in 10 studies (Table 3); Eg101 was used in 4 studies, Israel 1998 (IS-98-ST1) in 3 studies, and AR 381/00, B956, Morocco 96.111, and NY99 were each used in one study. One study used both lineage 1 and 2 viruses (Eg101 and B956, respectively), while one study used a virus belonging to genetic lineage 2 (AR 381/00) only. Two studies did not specify the virus used in the neutralization test. One study used Ib-AN 7019 for the complement fixation test.

Although the majority of the studies (*n* = 10) did not screen further for neutralizing antibodies to other flaviviruses, Usutu virus (USUV) was the most investigated, with USUV only investigated in 3, USUV and JEV in 1, and USUV and tick-borne encephalitis (TBEV) in 1 (Table 3). The South African strain of USUV, SAAR-1776, was used to detect neutralizing antibodies in three studies while one study used the recombinant E domain III (rEDIII) of USUV (USUV.EDIII) and TBEV (TBEV.EDIII). One study did not mention the strain of virus used in PRNT to detect JEV-specific neutralizing antibodies. Other flaviviruses investigated included: yellow fever virus, Wesselsbron virus, Potiskum virus, and Uganda S virus. Positive titers were detected against USUV and undetermined flaviviruses in 2 and 1 studies, respectively. In one study, results of multiplex immune-assays and micro-neutralization tests differed for 5 samples; 2 samples were positive for WNV and USUV, 2 were positive for USUV and 1 undetermined by MIA while all 5 were negative for USUV by MNT and the undetermined flavivirus was WNV positive. In one study, complement-fixing antibodies were detected against yellow fever virus, Wesselsbron virus, Potiskum virus and Uganda S virus.

**Figure 2:**
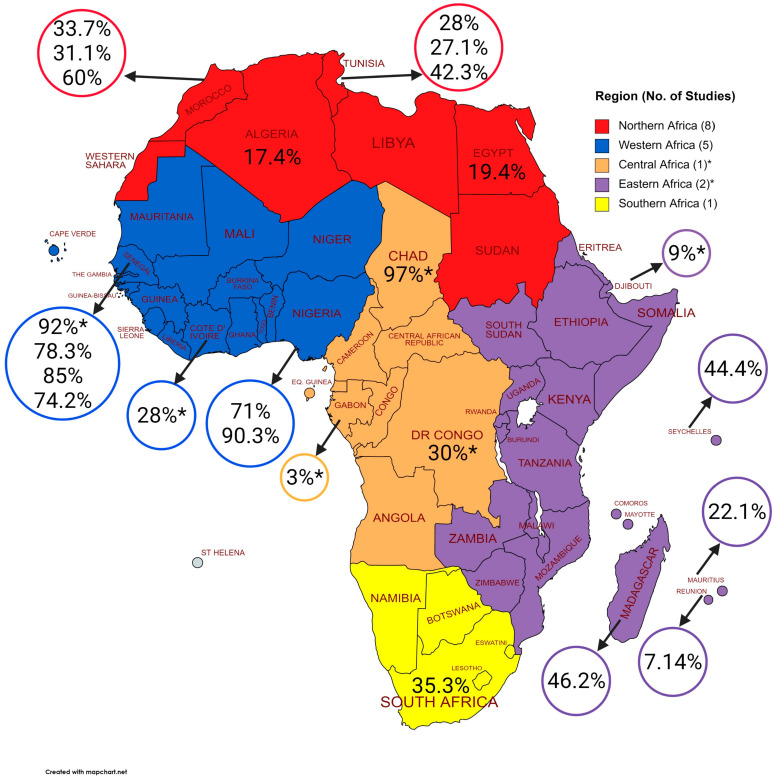
Map of Africa showing seroprevalence reported in different countries (* indicates study where samples were screened from multiple countries [31]). Created with MapChart.net (accessed on 26 May 2021).

In total, 1998 equids (35%) were positive for WNV antibodies out of the 5746 animals screened in Africa. The seroprevalence reported by studies ranged from 17.4 to 90.3% (Table 3). However, although Cabré et al. [31] reported an overall seroprevalence of 36% for samples collected from six countries between 2002 and 2005, this ranged from 3% in Gabon in Central Africa to 92% in Senegal in Western Africa. The prevalence in Northern Africa ranged from 17.4% in Algeria to 42.3% in Tunisia, while in Western Africa, it ranged from 35.9% in Nigeria to 85% in Senegal. In Southern Africa and sub-Saharan Africa, a prevalence of 35.3 and 35.9%, respectively, was reported (Figure 2). There was no apparent trend in seroprevalence over time. The earliest study [39] found a seroprevalence of 71% in Nigeria in 1987 whereas the most recent study [40] found a seroprevalence of 19.4% in Egypt in 2019. Similarly, two independent studies conducted in 2014 in Algeria and Senegal had a seroprevalence of 17.4% and 74.2%, respectively [35,38].

In 4 of the 16 studies, longitudinal samples were obtained from 615 equids and seroconversion was observed in 12% of them. In one study [37], seroconversion was observed in 50 equids (11.1%) that became seropositive as yearlings after testing negative as foals. A further 4 seroconverted a year after the first screening. More of the foals born earlier in the foaling season (August to December) seroconverted than those foaled later [37]. In Tunisia, of 84 horses that initially tested negative, 13 were lost to follow-up and only 2 (2.4%) seroconverted [28]. In one study, 36 horses (18 each in Chad and Côte d’lvoire) were sampled again one year later [31]. In Chad, 2 of 3 horses that initially tested negative were positive and 5 of the 15 initially positive horses were no longer positive. In Côte d’lvoire, none of the eight initially negative horses had seroconverted a year later and only one of ten horses remained positive. In one study [38], two horses became seropositive 8 months after arriving in El Kala, Algeria.

### 2.4. Risk Factors

Risk factors for seroprevalence of WNV among the equids were assessed by 13 studies. Risk factors may be host dependent (e.g., species, breed, age, sex) or non-host dependent (e.g., environmental). Ten studies discussed both host and non-host dependent factors in relation to the seroprevalence and three [30,31,40] discussed only non-host dependent factors.

In one study, a significant difference between seroprevalence of horses and donkeys was identified [38]. In another study, breed-specific seroprevalence of WNV varied significantly with a higher likelihood of a local breed, Dongola, being seropositive than the imported Argentine [41]. WNV seroprevalence increased with age [37]. This increase was reported to be significant in several studies [32,33,34]. Cardinale et al. [32] reported odds of infection about three times higher in horses aged 11–15 years than those aged 1–5 years. Similarly, Guthrie et al. [37] found the odds of being seropositive were about 5 times higher for older mares (aged 10–22 years) compared to younger mares (aged 6–9 years). Being a stallion was a protective factor [36]. As mentioned above, in a study conducted in South Africa, foals born in August–October 1999 had a higher risk of seroconverting in their first year of life than those born November–December 1999 [37]. Furthermore, those born to seropositive mares had about half the risk of seroconverting of those born to seronegative mares [37].

In a study conducted in Senegal, Chevalier et al. [34] randomly sampled equids at five locations with different landscape types. In a statistical model that included equid age, they found that lower seroprevalence was associated with areas with saline water and arid salty mud flats, environments that are unfavorable for *Culex* mosquito larvae [34]. Similarly, WNV infection did not occur in equids from coastal lowlands in contrast to a widespread infection in Highveld/plateau and Western Cape Province regions of South Africa [37]. Other studies reported significant differences in seroprevalence in horses in different locations in Northeast Algeria and Morocco [36,38]. Only four studies described the mobility of the equids sampled [27,31,32,38].

## 3. Discussion

In this scoping review, 16 studies were identified in which authors described the seroprevalence of WNV among African equids. Two recently published studies [42,43], were outside our search period. An article referenced in one of these articles was not found in our search [44].

Overall, this review revealed an uneven spread of research on WNV seroprevalence across the five regions of Africa. More studies were reported in North and West Africa with only one report in Southern and Eastern Africa, while another study had equid representation from central and Eastern Africa. East Africa, the origin of the virus [2], is under-represented despite the burden of the disease in the region [45,46,47]. The two studies that involved the region are not a true representation of WNV seroprevalence among equids in the region as only animals from Djibouti [31] and four Indian Ocean islands [32] were sampled. Africa has a wide range of climatic conditions. The overall seroprevalence reported by Cabré et al. [31], who screened samples obtained from East, Central and West Africa, was 36% but there was a wide range of seroprevalence for individual countries. The highest seroprevalence (92–97%) was observed in the western and central parts of the Sahelian zone, which is typified by semi-arid climate and steppe and bush grass vegetation. In contrast, the sub-Sahelian zone, with an arid climate and a semidesert vegetation had lower seroprevalence (3–30%). A regional difference in seroprevalence was also observed in equids sampled from five unique bioclimatic regions of Morocco in 2011 [30]. However, other factors can influence seropositivity. The significant relationship between WNV seropositivity in a study in Northeast Algeria can be linked to the arrival of equids in a town bordering Tunisia where they seroconverted [38]; Tunisia already had WNV circulating in 2014 [48].

There was considerable heterogeneity in the design of the 16 reviewed studies. Although expensive to execute, probability sampling is more reliable with a representative population than non-probability sampling techniques. An increased sample size following an appropriate determination of the minimum sample size is often used to improve reliability of the latter. The non-calculation of the minimum sample size in most studies may explain the wide range (62–1189) in the total number of equids sampled across all studies.

All except two of the studies [37,39] employed ELISA as a preliminary screening assay. Mostly, the species-independent ID-Vet ELISA was used for the detection of the WNV anti-envelope (E) protein along with a few in-house ELISAs, even though some immunofluorescent-based tests are also commercially available. Despite the benefit of a quick and reproducible result, cross-reactivity between flaviviruses remains a serious limitation for using ELISAs or immunofluorescent tests alone. ELISA alone often results in false positives due to co-circulation of diverse flaviviruses [49]. Neutralization tests can resolve discrepancies that often occur with other assays due to cross-reactivity. Confirmatory neutralization tests were performed in most studies after initial screening with ELISA, microsphere immunoassay [26] or Western blot [31]. Neutralization tests performed included MNT, VNT and PRNT. Not paying attention to the cross-reactivity of flaviviruses among African equids could result in an erroneous representation of the seroprevalence of the disease. For instance, equids positive for USUV, USUV and WNV and undetermined flavivirus based on MIA were all confirmed as WNV positive by virus neutralization test [26]. On the other hand, one of the studies showed that seroprevalence determined by neutralization test varied only slightly from that determined by ELISA (30.4% vs. 33.7%, respectively) among equids in Morocco [26]. The survey in Tunisia [28] might have used ELISA alone based on the fact that two WNV epidemics had been reported before the survey [12,50]. This, however, does not fully account for co-circulation of other flaviviruses. Another approach that provides some reassurance that antibodies are specific to WNV is to screen vectors for the presence of virus in parallel. A recently published article screened 368 horses in Nigeria using ELISA alone along with 775 adult female *Culex* mosquitoes (pooled in 31 groups) from the stables of sampled horses. The resultant 89.9% seroprevalence in horses was in stark contrast to detection of the presence of WNV antigen in only one pool of mosquitoes using the VectorTest^®^ WNV Antigen Assay [42]. Similarly, Assaid [26] found that 33.7% (31/92) of horses were antibody positive, but only one out of 146 mosquito pools was positive for WNV. This differed from the study in Egypt included in this review in which 500 horses and 5 pools of Culex mosquitoes (100 per pool) were sampled with an equine seroprevalence of 19.4% and the detection of the NS5 gene by RT-PCR in one pool (i.e., 20%) [40]. A recent study in Tunisia where a sheep serum sample was confirmed to be TBEV seropositive [51] together with reports of undetermined flavivirus antibodies in some of the studies from North Africa [26,29,36] suggest that antibodies to TBEV also need to be differentiated, at least in North Africa.

Demographic data (age, sex, breed, species) of the equids sampled are important in determining specific seroprevalence for different strata in the population and the risk factors associated with seropositivity. Although most studies reported the age of the equids, a few had no age description making it difficult to compare age outcomes across all studies. Two studies [27,38] described equids as ‘adults’ without defining this category, making assessment across age groups not possible. Nonetheless, several studies identified a significant increasing risk of seropositivity with age. The rise in age-specific seroprevalence of WNV observed as equids increase in age might be indicative of endemic transmission of WNV. The breeds of equids sampled were seldom mentioned [33,37,39,41] and any breed-specific differences in seroprevalence were difficult to determine. This challenge might be because of the diversity of horse breeds in Africa coupled with importation of several breeds that are often crossed with indigenous breeds. This probably influenced the description ‘different’ and ‘various’ breeds by some authors [26,30]. Categorizing breed types either as draft horses (used for work), light horses (for riding), crosses or ponies where specific breeds cannot be determined could reveal a general trend in disposition to WNV seropositivity. Polymorphisms in the innate immune Oas1b gene have been identified as a determining factor for resistance to WNV [52]. However, to date, no difference in susceptibility to WNV infection between major equine breeds in the United States has been reported. Not all the studies described the sex of the equids sampled. Being a stallion was a protective factor [36], but this could be related to stallions being housed differently (e.g., more likely to be stabled).

There are four WNV vaccines available commercially for equids, three in the United States and one in Europe [53,54]. Vaccination of horses may not be common in Africa as it is probably limited to valuable sporting horses, but horses imported as adults could be previously vaccinated. It is therefore paramount that the WNV vaccination status of horses in Africa be declared when enrolled in seroprevalence studies. When this status is unknown, it should be stated clearly with the origin of the horse mentioned. In the study in this review where all sampled equids had been vaccinated against WNV (>400 days before sampling and most had received a single dose in 2010), it was presumed that vaccine antibodies waned (detected up to 300 days post injection). An ELISA to detect anti-NS1 antibodies was used in this study to differentiate infected from vaccinated horses, with 79% of the cELISA positive sera also positive for WNV NS1 antibodies indicative of infection. Conflicting results between cELISA and NS1 could suggest infection with a different flavivirus as two NS1 negative horses were found to have antibodies to USUV [36].

In one of the studies reviewed here, military working dogs in Morocco showed similar seroprevalence (62%) to that seen in horses (60%) leading to the suggestion that they could be an alternative sentinel species [36]. In contrast, another reviewed study [35] found that seroprevalence in dogs was less than half that in horses (27.3% vs. 68.7%). This study also found that seroprevalence was low in goats (6.9%) and no antibodies were detected in sheep or cattle. This agrees with a report from southern France where some animals including cows, goats, camels and zebra, tested following an outbreak among horses, were all negative [55]. Similarly, on the island of Corsica, France, sheep sera tested negative while seroprevalence in horses and dogs was 9.4% and 8.4%, respectively [56]. There have also been reports in China of WNV-neutralizing antibodies circulating in dogs and even cats [57] and 24 of 183 hunting dogs (13.11%) sampled in Italy recently showed seroneutralizing antibodies to USUV, but no antibodies to WNV were detected [58]. Thus, the horse remains the most relevant species to monitor circulation of WNV, despite the potential complication of vaccine-induced antibodies. Interestingly, in one article [29], it was reported that the presence of cattle is a protective factor. Seroprevalence of WNV among resident and migrating species of bird in northern Senegal [59] corroborates the high seroprevalence observed in equids in the region [33]. Despite several reports of WNV epidemics in Sudan [60,61,62] and a recent detection of the virus from wild mosquitoes in Khartoum, there are no prevalence reports among equids. In Nigeria, where the virus was isolated from humans in 1973 [63], it has become an increasingly-recognized public health issue across this most populous African nation [64,65].

In this scoping review, we observed the relative dearth of WNV seroprevalence studies in African equids (5746 equids screened in 16 studies published between 1937 and 2020) across all regions of the continent despite the early WNV outbreaks and seropositivity in humans and animals [66,67,68,69,70]. The low numbers of clinical cases of WNV among both human and veterinary species that have been reported could be blamed on misdiagnoses and lack of funding in the region [71]. Only a few seroprevalence studies of WNV among equids were reported before 2010 [31,33,37,39] and, apart from the report by Cabré et al. [31], were geographically limited. Although the number of publications increased from 2010 onward, and two studies published after the literature search date were conducted in West Africa [42,43], this was still minimal compared to a review of WNV seroprevalence studies in Europe [72] that identified 39 studies published between 2001 and 2018 involving a total of 14,327 equids.

Despite speculation that climate change is leading to increased circulation of vector-borne viruses such as WNV, there was no discernible trend in seroprevalence across the years covered by the studies reviewed. Levels of seroprevalence were likely to be strongly influenced by location, timing of sampling, and other factors. For example, for two studies conducted in the same year, a seroprevalence of 17.4% was reported in Algeria [38] and 74.2% in Senegal [35]. The occurrence of a WNV outbreak may prompt subsequent seroprevalence studies (e.g., the study by Benjelloun et al. [30] was conducted the year after an outbreak in 2010).

Some studies were evidently performed in the rainy (wet) season when mosquito vectors are more abundant, while some were conducted over a longer period. The abundance of mosquito vectors suggests increased transmission and more infection. This might be the reason for the very high seroprevalence observed in equids sampled in the wet season in Senegal (78.3% and 85%) and Nigeria (90.3%) [33,34,41]. A high seroprevalence, 74.2%, was also observed among equids sampled at the end of the dry season [35]. It is challenging to describe the climate of the sampling period for studies that reported sampling period based on calendar month (January–December). With climate change, it is often difficult to describe the climate based on the calendar month, especially for Africa.

As equids are often used as draft animals and also in sport or leisure, the seroprevalence rate may indicate human exposure to WNV. The publication date of some of the studies was much later than the period the research/investigation was conducted. For instance, two studies conducted in 2010 and 2011 were both published in 2017 [30,32] while another conducted in 2012 was published in 2016 [35]. This time lag impacts the real-time comprehension of the status of WNV as well as the risk, thus impairing surveillance and public health responses. Regional and sub-regional collaborations could help reduce the period between field sampling and publication. Although a wide range of seroprevalence was reported, making it difficult to have a single reliable seroprevalence value for the different regions and individual countries in Africa, the high seroprevalence in some regions is a call to institute a workable regional or sub-regional surveillance protocol for Africa. One reason for the few numbers of studies across some regions could be the expense of the commercially available ELISA kit, coupled with the difficulty of confirmatory assays that need to be performed in CL3 laboratories, suggesting that development of cheaper but reliable and specific antibody tests are required. Furthermore, a regional collaborative research could ameliorate the deficit and encourage a One Health strategy to tacking the burden of WNV in Africa.

## 4. Materials and Methods

### 4.1. Search Strategy

The search strategy aimed to identify relevant published studies. Three electronic databases, PUBMED (Medline), OVID (via CAB Abstracts, Embase and Zoological records) and African Journal Online (AJOL) were searched to identify peer-reviewed articles on the topic. The literature search was performed using the key terms and Boolean operators (‘AND’ and ‘OR’) as follows: (Seroprevalence OR prevalence OR epidemiology OR frequency OR occurrence OR detection OR report OR identification OR isolation OR characterization OR investigation) AND (West Nile virus OR WNV OR Flavivirus) AND (Horses OR Equine OR Donkey OR Equid OR Mule OR Foal OR Zebra) AND (Africa OR South Africa OR West Africa OR East Africa OR North Africa). The search terms were applied to all fields with no language restrictions. Retrieved hits were entered into a Microsoft Excel (2019) file. The reference lists of all studies selected for critical appraisal were screened to expand the search.

### 4.2. Eligibility Criteria

Peer-reviewed studies published between 10 July 1937 and 10 July 2020 conducted in any of the 54 countries of the African continent were screened for eligibility. The following studies were considered eligible: primary research, descriptive and cross-sectional studies, and prospective and retrospective studies. Analytical observational studies, including longitudinal cohort studies and analytical cross-sectional studies, were considered for inclusion. Case reports, case series and reviews were considered ineligible. This review considered prevalence determined by laboratory assessment of antibodies to WNV. The eligible study population was equids (horses, donkeys, mules and zebra) of all ages. Conference abstracts and reviews were not included in this scoping review.

### 4.3. Study Selection

Firstly, O.T.O. and J.M.D. screened all search results and removed duplicates before an independent screening of titles and abstracts of all retrieved studies for relevant potential studies. All articles with titles and abstracts not fulfilling the eligibility criteria above were excluded, along with studies that had missing or duplicated data published in included studies. Full texts were screened when eligibility could not be ascertained from the title and abstract. Secondly, all full texts retained were scanned independently in a standardized approach by O.T.O. and J.M.D. using the eligibility criteria. Only studies with accessible full texts were retained and subjected to eligibility checks.

Following completion of eligibility evaluation, the full text of selected citations were assessed in detail by two independent reviewers (O.T.O. and J.M.D.). Reasons for exclusion of full text studies that did not meet the inclusion criteria were recorded and reported.

### 4.4. Data Extraction

A custom data extraction form was created with data categorized into: general data (first author’s name, year of publication, title, name of journal, language, country, region, study type, sampling protocol, etc.), diagnostic technique data (initial serological screening for immunoglobulin G, type of confirmatory test and other additional tests performed) and data related to the animal (species of equid, number of equids, sex, breed, WNV vaccination history, seroconversion, season of investigation and risk factors investigated). Disagreements that arose between the reviewers O.T.O. and J.M.D. were resolved through discussion, or by a third reviewer (M.B.).

## 5. Conclusions

This scoping review highlights the dearth of seroprevalence studies among African equids. WNV is endemic among equids in all regions of Africa and the risk of infection increases with age. Being good sentinels for WNV, there is a need for a subregional and regional collaboration targeted towards WNV among equids in Africa, thus curbing the threat of WNV to humans in Africa. ELISAs, Western blot and neutralization tests are established diagnostic methods in Africa. Researchers should employ specific serological tests to rule out possible cross-reactivity with other flaviviruses. For future research, detailed description of demographics of equids and associated risk factors to WNV and other related flaviviruses among African equids is encouraged. A ‘One Health’ initiative involving governmental and non-governmental parastatals along with other stakeholders in ministries, departments and agencies should be established to come up with a national, sub-regional and regional strategic plans for routine surveillance and reporting of WNV among equids and other animals as a public health preparedness plan to curb this zoonotic disease. These are very pressing issues that require a collaborative regional One Health approach [71].

## Figures and Tables

**Table 1:** Summary of study characteristics of the 16 included studies.

Study	Study Year	Country/ies ^1^	Region	Study Design	Sampling Technique
Assaid 2020 [26]	NS	MA	North	Descriptive	Convenience
Bargaoui 2015 [27]	2009	TN	North	Cross-sectional	Multistage (Random)
Ben Hassine 2011 [28]	2008	TN	North	Cross-sectional	Cluster
Ben Hassine 2014 [29]	2012	TN	North	Cross-sectional	Random
Benjelloun 2017 [30]	2011	MA	North	Cross-sectional	Convenience
Cabré 2006 [31]	2002–2005	DJ CG, GA, TD BJ, CI, SN	East Central West	Cross sectional	Convenience
Cardinale 2017 [32]	2010	MG, MU, RE, SC	East	Cross-sectional	Convenience
Chevalier 2006 [33]	2003	SN	West	Cross-sectional	Convenience
Chevalier 2010 [34]	2005	SN	West	Cross-sectional	Convenience
Davoust 2016 [35]	2014	SN	West	Cross-sectional	All available
Durand 2016 [36]	2012	MA	North	Cross-sectional	Judgmental/ Purposive
Guthrie 2003 [37]	2000–2001	ZA	South	Cohort	Judgmental/ Purposive
Lafri 2017 [38]	2014	DZ	North	Cross-sectional	Convenience
Olaleye 1989 [39]	1987	NG	West	Cross-sectional	Convenience
Selim 2020 [40]	2019	EG	North	Cross-sectional	Judgmental/ Purposive
Sule 2015 [41]	2011–2012	NG	West	Cohort	All available

NS, not specified. ^1^ Two-letter ISO code: BJ, Benin; CG, Republic of the Congo; CI, Cote d’Ivoire; DJ, Djibouti; DZ, Algeria; EG, Egypt; GA, Gabon; MA, Morocco; MG, Madagascar; MU, Mauritius; NG, Nigeria; RE, Reunion; SC, Seychelles; SN, Senegal; TD, Chad; TN, Tunisia; ZA, South Africa (ZA).

**Table 2:** Summary of population demographics from 16 studies.

Study	N	Species (n)	Breed(s)	Age	Sex
Assaid 2020 [26]	92	Horse	Mixed	>2 years	M, F
Bargaoui 2015 [27]	1189 ^1^	Horse (272) Donkey (807) Mule (107) NS (2)	NS	Adults	NS
Ben Hassine 2011 [28]	133 ^1^	Horse (23) Donkey (93) Mule (17)	NS	NS ^2^	NS ^2^
Ben Hassine 2014 [29]	284	Horse (28) Donkey (219) Mule (37)	NS	2–18 years	M, F
Benjelloun 2017 [30]	840	Horse	Mixed	>3 years	M, F
Cabré 2006 [31]	245	Horse	NS	NS	NS
Cardinale 2017 [32]	303	Horse	NS	Mostly adults	M, F
Chevalier 2006 [33]	120	Horse	Local	1–21 years (7.5) ^3^	NS
Chevalier 2010 [34]	367	Horse	NS	2–24 years	NS
Davoust 2016 [35]	283	Horse (64) Donkey (29) Sheep (136) Goat (29) Cattle (14) Dog (11)	NS	Adult	NS
Durand 2016 [36]	580	Horse (349) Dog (231)	NS	1.5–32 years (14.5)	M, F
Guthrie 2003 [37]	731	Horse	Thoroughbred	Foal: 2.3–10.2 months (6.6) ^4^ Yearling 15.2–20 months (18.1) ^4^ Dam: 6–22 years (11) ^4^	NS for foals/yearlings
Lafri 2017 [38]	293	Horse (71) Donkey (222)	NS	Adult	M, F
Olaleye 1989 [39]	62	Horse	Dongola and Arab-Barb	NS	M
Selim 2020 [40]	500 ^1^	Horse	NS	NS	NS
Sule 2015 [41]	145	Horse	Argentine and local Dongola	≥3 years	M, F

^1^ Sample size calculation performed; ^2^ Information on age and sex was collected in a questionnaire but not reported; ^3^ Mean age; ^4^ Median age. Abbreviations: NS, not specified; M, male; F, female.

**Table 3:** Summary of serological tests and seroprevalence outcomes from 16 studies.

Study	Test	WNV Used in NT	Other Flaviviruses	Seroprevalence % (CI)
Assaid 2020 [26]	ELISA ^1^, MIA, MNT	IS-98-STI	TBEV, USUV	33.7
Bargaoui 2015 [27]	ELISA ^1^, MNT	IS-98-STI	-	28
Ben Hassine 2011 [28]	ELISA ^1^	-	-	27.1
Ben Hassine 2014 [29]	ELISA ^1^, PRNT	Eg101	USUV	42.3
Benjelloun 2017 [30]	ELISA ^1^, VNT	Morocco 96.111	-	31.1
Cabré 2006 [31]	ELISA, WB, PRNT	Eg101	-	36
Cardinale 2017 [32]	ELISA ^1^, PRNT	NY99	JEV, USUV	27.39
Chevalier 2006 [33]	ELISA, PRNT	NS	-	78.3 (64.0–92.7)
Chevalier 2010 [34]	ELISA, PRNT	B956 and Eg101	USUV	85 (81–89)
Davoust 2016 [35]	ELISA, WB	-	-	74.2
Durand 2016 [36]	ELISA ^1^, VNT	IS-98-STI	USUV	60 (55–65)
Guthrie 2003 [37]	VNT	AR 381/00	-	35.3
Lafri 2017 [38]	ELISA, WB, PRNT	Eg101	-	17.4
Olaleye 1989 [39]	CFT	Ib-AN 7019	POTV, UGSV, WESSV, YFV	71
Selim 2020 [40]	ELISA ^1^	-	-	19.4
Sule 2015 [41]	ELISA ^1^, PRNT	NS	-	90.3 (84.3–94.6)

^1^ Commercially-available IDvet ELISA used. Abbreviations: CI, confidence intervals; CFT, complement fixation test; ELISA, enzyme-linked immunosorbent assay; JEV, Japanese encephalitis virus; MIA, multiplex immune-assay; MNT, micro-neutralization test; NS, not specified; POTV, Potiskum virus; PRNT, plaque reduction neutralization test; TBEV, tick-borne encephalitis virus; UGSV, Uganda S virus; USUV, Usutu virus; VNT, virus neutralization test; WB, Western blot; WESSV, Wesselsbron virus; YFV, yellow fever virus.
